# Inter-evaluator and Intra-evaluator Reliability of a Software Program Used to Extract Kinematic Variables Obtained by an Extremity-Mounted Inertial Measurement Unit System in Sound Horses at the Trot Under Soft and Hard Ground Conditions and Treadmill Exercise

**DOI:** 10.3389/fvets.2021.595455

**Published:** 2021-03-04

**Authors:** Julia Schwarz, Beatriz Vidondo, Ugo E. Maninchedda, Miriam Sprick, Melina C. Schöpfer, Antonio M. Cruz

**Affiliations:** ^1^Vetsuisse Faculty, Institut Suisse de Medicine Equine, University of Bern, Bern, Switzerland; ^2^Vetsuisse Faculty, Veterinary Public Health Institute, University of Bern, Bern, Switzerland; ^3^Clinic of Equine Surgery, Faculty of Veterinary Medicine, Justus-Liebig-University Giessen, Giessen, Germany

**Keywords:** reliability, gait, horse, surface, IMU, kinematics

## Abstract

**Objective:** To assess the inter-evaluator and intra-evaluator reliability of a software program used to extract kinematic variables by a commercially available extremity-mounted inertial measurement unit system in sound horses at the trot under soft and hard ground conditions and treadmill exercise.

**Animals:** Thirty adult, sound and healthy French Montagne stallions.

**Procedures:** Data collection was performed with six IMUs strapped to the distal, metacarpal, metatarsal and tibial regions of every horse. Per surface (treadmill, soft and hard ground) 10 stallions were trotted three times. Prior to the analysis done by six evaluators (three experienced, three inexperienced) the data was blinded and copied three times. For every analysis a minimum of five strides had to be selected. To assess the intra- and inter-evaluator reliability a selection of gait variables was used to calculate intra and inter correlation coefficients (ICCs) as well as variance partitioning coefficients (VPCs).

**Results:** All of the tested gait variables showed high levels of reliability. There was no mentionable difference considering the correlation coefficients between the intra and inter reliability as well as between the three different surfaces. VPCs showed that the factor horse is by far the most responsible for any appearing variance. The experience of the evaluator had no influence on the results.

**Conclusions and Clinical Relevance:** The software program tested in this study has a high inter- and intra-evaluator reliability under the chosen conditions for the selected variables and acts independent of the ground situation and the experience of the evaluator. On the condition of a correct application it has the potential to become a clinically relevant and reliable gait analysis tool.

## Introduction

Visual lameness examination is one of the most common tasks carried out by equine veterinarians and relies largely upon the experience and expert knowledge of the veterinarian. It has been shown to be a subjective and highly variable method (1, 2). In the other hand, electronic lameness detection systems commercially available try to overcome this deficiency by accurately and objectively capturing gait events that the human eye is unable to do with similar precision (2, 3).

Over the last years the number of commercially available portable electronic systems capable to detect some aspects of equine gait has increased. Due to their reduced size and simplicity in utilization they have become clinically applicable and expensive research laboratories are no longer the only option to perform gait analysis and lameness detection (4).

Inertial measurement units (IMU) consist of a combination of accelerometers and gyroscopes and often magnetometers. They are able to derive acceleration, orientation and indirectly velocity (5). The field of application is widely spread and range from navigation systems and control of unmanned ground vehicles or aerial systems (5) to motion analysis (6) and control of artificial limbs (7).

In studies of human motion analysis the use of IMUs is common and can offer an accurate and reliable method (6). Among others they enable measurements of joint angles (8), motion of the lumbar spine (9) and head movement (10). In the equine field IMUs are often used for gait analysis and to assess lameness through measurements of either head and back movements (11) or motion of limbs (12). Some of these systems have been extensively tested prior to clinical implementation (11, 13, 14). Specific studies have already been performed and proved a good repeatability and practical application of some electronic lameness detection systems for clinical use (11–13, 15, 16).

The extremity-mounted IMU system tested in this study has a patent for the application in horses as well as in humans (17). Extremity mounted sensors used in this study are capable to provide spatiotemporal variables of extremities and allow the analysis of extremity motion in horses (6, 15).

In this study extremity mounted IMUs determine limb phasing as the basic output variable to determine each gait cycle. Limb phasing has been described in horses and humans (6, 18, 19) and conceptually described initially by Hildebrand based on the Muybridge experiments (20). Limb phasing is based on the concept that each limb displays a similar cyclic or sinusoidal motion and determines the temporal relationships between the respective limbs through signal processing and a cross-correlation approach (18–20).

As the data output can be sent electronically to an experienced analyst to remotely interpret the data of a particular horse, in addition to repeatability, inter and intra- reliability studies are important to prove that a particular system's output is independent of the software operator, a feature that is considered critical and highly desirable for the clinical application of any measuring tool.

Therefore, the objective of this study was to assess the inter- and intra-evaluator reliability of a commercially available software analysis program (Poseidon 9.0, European Technology for Business Ltd, Hitchin SG4 8WH, UK) used to extract the data provided by an inertial measurement unit system in non-lame horses at the trot under different surface conditions.

## Materials and Methods

### Ethical Considerations

The experiment was approved by the Animal Health and Welfare Commission of the Canton of Vaud and followed institutional guidelines for humane animal treatment (approval number VD3087; date of approval 11 February 2016).

### Subjects

Thirty healthy adult Franches-Montagne stallions of similar size and mass were randomly selected out of a herd at the Swiss National Stud Farm in Avenches, Switzerland. Stallions were evaluated to be sound and healthy based on a thorough clinical examination by a qualified veterinarian. All horses used in this experiment were regularly shod every 6 weeks using regular open shoes by a professional blacksmith. The horses were between 6 and 19 years old, had a body weight of 537.5 ± 28.3 kg and a height at withers of 157.4 ± 1.4 cm. Horses were in good physical condition, disease and medication free and got exercised daily. The data collection with the IMUs was performed in an environment which was familiar to the horses and they were all accustomed to work on the treadmill.

Horses were exercised in three different surfaces. For every surface 10 horses were selected for the data collection.

The soft surface consisted of a geotextile polymer mix (Terra-Tex, Terra-Bausysteme GmbH, 77743 Neuried, Germany) and was located in a closed arena. The dimensions of the arena were 44 m in length, 24 m in width and 50 m on the diagonal.

A straight path of crashed rock of 100 meters length was used to exercise horses under hard surface conditions.

The treadmill (Mustang 2000, Kagra, Graber AG, Fahrwangen, Switzerland) was calibrated prior the data collection using a magnetic speedometer system (Anima +, TwoNav, Arenys de Mar, Barcelona, Spain).

### Inertial Measurement Units (IMUs)

The IMUs (Pegasus GaitSmart, European Technology for Business Ltd, Hitchin SG4 8WH, UK) which were used in the study have a total weight of 54 g and dimensions of 73 x 36 x 19 mm per sensor. Furthermore, a patented software (Poseidon version 9.0 European Technology for Business Ltd, Hitchin SG4 8WH, UK) and a laptop were included in the IMU sensor system. According to the US patent (17) each IMU contained a tri-axial 5 g accelerometer and three single axes, 1,200 deg/s gyroscopes followed by anti-aliasing filters with a cut-off frequency of approximately 50 Hz. This combination allows it to record 6 degrees of freedom, consisting of angular velocity and acceleration along the three orthogonal axes. A 12 bit analog-to-digital converter was responsible for the data sampling at a frequency of 102.4 HZ. To achieve a relative drift, which is <10 ms/h after the synchronization between the single units, the IMUs were factory set within 1 ppm (=3.6 ms/h) of a reference time. At the beginning of every trial each IMU was time stamped and synchronized by sending a simultaneous pulse to each unit by using the specially written software. The collected data was saved on an internal memory storage service card in the IMU and was later transferred to a computer by using the software and connecting the sensors to the computer (12).

**Figure 1:**
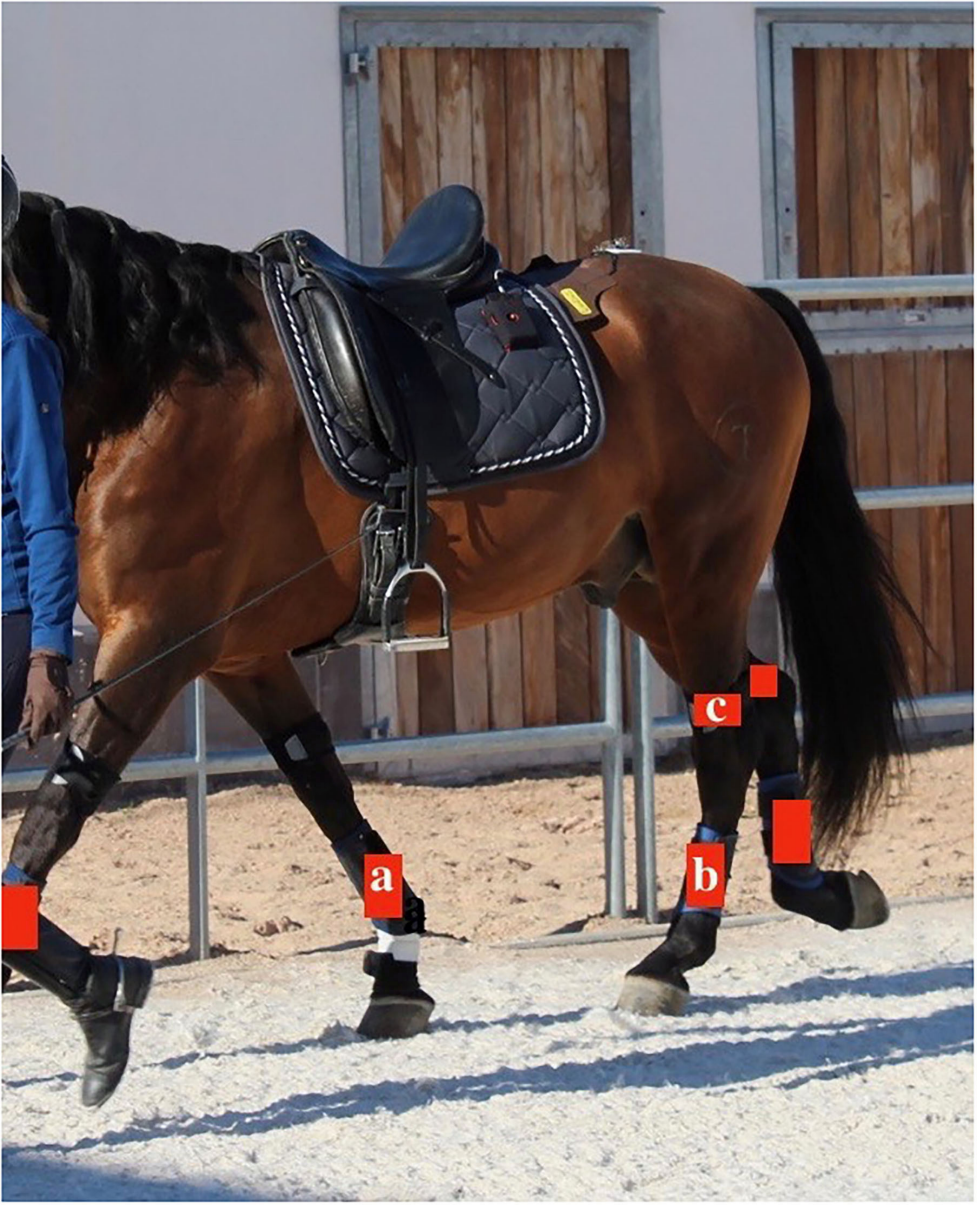
Placement of the sensors (red). On the front limbs the sensors were placed on the distolateral aspect of the metacarpal region **(a)**. On the hind limbs the sensors were placed on the distolateral aspect of the metatarsal region **(b)** and on the distolateral aspect of the tibia in the groove just dorsal to the calcaneal tendon **(c)**.

For the data collection six sensors were used on each horse. With the help of brushing boots (Woof Wear, Bodmin, Cornwall, England) which were modified with a small pouch it was possible to secure the sensors on the distolateral aspect of the metacarpal and metatarsal regions on each of the four extremities. The remaining two sensors were fixed on the distolateral aspect of each tibia in the groove just dorsal to the calcaneal tendon with a two custom-made soft straps that included as well a small pouch (Figure 1). This construct enabled to capture motion measurements of each cannon and the hock joints (17).

### Data Collection

Immediately prior to data collection and in preparation for it, all horses underwent identical warm-up procedures described somewhere else as this study took part of the data from a previous one (12).

IMUs were synchronized and time stamped using the associated software. To secure the sensors at a standard location the horse was equipped after the warm up with the brushing boots and straps as described above. Because the placement of the boots and straps may influence the movement of the horses the horses were walked and trotted until the gait appeared visually normal. Then the sensors were placed in the small pouches and switched on followed by a stand still period of 10 s to enable the sensors to define the gravitational vector and to calibrate.

Disturbing factors which could have affected the behavior and movement of the horse were eradicated as effectively as possible.

For the soft surface the horses were trotted in the arena on the diagonal (50 m) at their natural speed (3.51 ± 0.33 m/s) which was measured with a chronometer over a 10 meter distance.

The measurements for the hard surface took place outdoors, a minimum of 20 strides were collected at the horses natural speed on a straight line. Speed for the hard surface trials was not collected.

For the data collection on the treadmill the speed was set to 3.3 m/s and the horses were trotted a minimum of 20 strides.

During the data collection each horse was trotted three times on every surface with a short walk phase in between trotting periods. A measurement was repeated if the horse was too excited or unfocused. These repetitions are important because data can only be visualized after the sensors are dismounted and connected to the laptop, which was done after every horse.

### Evaluators

The evaluators consisted of six people divided in two groups: experienced (three evaluators) and inexperienced (three evaluators). “Experienced” was defined by the prior completion of more than 100 analyses while the “Inexperienced” group had never worked with the system before. The inexperienced evaluators received instruction as to how to operate the software and then they performed the analysis independently. Both groups analyzed the same segment of trot but they may have chosen different data windows to analyze.

### Analysis Performed by the Evaluators

After downloading the collected data from the sensors to the laptop, the data had to be prepared for further analysis. Within every surface group the processed data of each horse was copied three times and blinded for the evaluators. This process delivered a total of 30 observations (10 Horses x three Repetitions) for each of the three surfaces, which were evaluated by each of the 6 evaluators.

**Figure 2:**
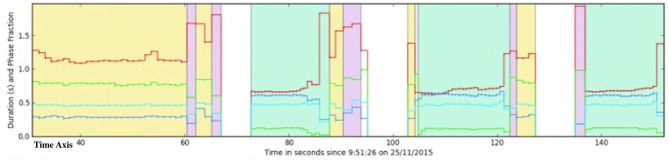
Example of a software screen where the evaluators had to visually select a time window within a preselected time segment for the purpose of this study. The preselected time segment always consisted of a trot phase (blue segment) and was specified for this study by an exact time frame using the time axis (selected segment lies between red arrows). In this screen, the entire trial is captured. Each yellow coded segment represents times when the horse was walking and each blue coded segment when the horse was trotting. The different color lines represent different aspects of the gait. For instance the red line represents the stride duration.

For the analysis the specific software on the computer (Poseidon 9.0, European Technology for Business Ltd, Hitchin SG4 8WH, UK) was used to process the collected data and display a temporal and orientation output. The evaluators had to visually select a data window within a preselected gait segment (Figure 2). This guaranteed that all analyses were made within the same gait segment. The objective was to select a minimum of five strides to be analyzed. On a steady state locomotion, characterized by a steady stride duration, seen in the graphic output of the system, the users can select a continuous segment of strides to analyse. From this selection, the system then works through a cross-correlation approach and selects the stride that is most representative by comparing each stride with each other doing minimal square difference calculations. The fewer strides available for selection, the higher the chances that the representative strides chosen will not be adequate. The selection of a minimum of five strides during steady state locomotion and with steady sensor signal is enough to ensure the resulting stride being representative of the horse's movement as 3–5 strides have been reported as the minimal number of strides needed for kinematic evaluation of horse's movement (21).

Areas with an irregular stride duration signal were avoided in the analysis such as the beginning and the end of the trot phase were the gait may not be representative due to acceleration or deceleration. No further instructions were given to the evaluators.

### Gait Analysis Variables

The IMU system is capable to collect information about more than 30 variables. Some of them are measured directly while others are calculated with the collected information. Basically they can be divided into two groups. On the one hand are the spatial variables which give information about different ranges of motions (ROM) and on the other hand the temporal variables which include data such as stride duration and limb phasing. Phasing is defined through a cross-correlation approach of the rotation velocity around the lateromedial axis of the inertial sensor, on a stride by stride basis, and is used to calculate the temporal phase-lag between respective limb cycles. Therefore, phase-lag is expressed as a percentage of the stride duration on a reference limb for each limb (18). This also allows for the automatic classification of gait.

The statistical analysis was performed only with a selection of variables (at least one variable of a subcategory). The evaluation of every single variable was not considered necessary because it is a repeated concept within a subcategory.

Selected temporal variables:

- Average stride duration in seconds.
- Limb phasing of the left and right forelimb and the right hind limb. Which are defined as the difference in time within a stride in percentage relatively to the set zero point, which is the point of maximal retraction of the third metatarsal of the left hind limb.
- Temporal variables were calculated as a percentage of the stride duration and included: Maximal metatarsal protraction and retraction for left and right and maximal metacarpal protraction and retraction for left and right.

Selected spatial variables:

- Sagittal range of motion (ROM): ROM of the left and right hock as well as the left and right fore cannon, measured in degrees.
- Coronal range of motion: medial-lateral movements of the fore and hind cannon for the left and right side, measured in degrees.
- Symmetry of the left and right hock in range of motion was calculated as the difference between the left and right limb values divided by the mean of both measurements.

### Statistical Analysis

The data was exported as a comma separated values and then imported into a spreadsheet (Excel, 2010, Microsoft Corporation, Redmond, USA). The blinding was unblinded for the statistical analysis. The temporal variable “maximal metatarsal protraction” was expressed as a percentage of the stride duration so the value of 98% is equal to 2%, 96% to 4%, and 94% to 6% and was recoded accordingly for the statistical analysis.

Then the data was imported into a commercial statistical software (NCSS 10, NCSS Statistical Software, Kaysville, Utah). The distributions of variables were all inspected using histograms and quantile plots and checked for Normality using Shapiro-Wilk and Kolmogorov-Smirnov tests (between other tests). Even though not always all tests showed evidence of Normality, all variables presented symmetric Gaussian-like histograms. Mean, median, standard deviation and interquartile ranges were calculated to describe the distribution of the variables. A repeated measurements ANOVA with evaluator as a within-subject factor was used to calculate mean squares that are in turn used to calculate the ICC and VPC. The general model repeated measures ANOVA was of the form:

$$\begin{array}{l} Y_{ijk}=\;\mu +\alpha_{i}+\;\beta_{j}+\;\varepsilon_{ijk} \end{array}$$

where *i* = 1,2,.,I, *j* = 1,2,.,J and *k* =1,2,.,K. Each surface was analyzed separately.

This model expresses the value of the response variable, Y, as the sum of the overall mean μ, the contribution of the *i-*th level of an explanatory variable α_*i*_ (= Evaluator ID, I = 6), the contribution of the *j-*th level of the subject variable β_*j*_ (= Horse ID, J = 10) and the contribution of the *k-*th (K = 3) individual measurement ε_*ijk*_ (often called error term, *n* = 180). Y were each of the final outcome variables presented in Tables 1–3.

**Table 1:** Mean/SD/IQR for each variable per surface.

	**Surface**		
**Variable**	**Treadmill**	**Soft**	**Hard**
No of strides evaluated	33.3/17.11/24.75	9.5/1.84/3	11.3/4.00/7
**Temporal variables**			
Stride duration (ms)	0.731/0.022/0.020	0.707/0.023/0.034	0.698/0.026/0.034
Limb phasing left forelimb (%)	63.097/0.733/0.955	65.547/1.394/2.193	64.292/2.222/2.441
Limb phasing right forelimb (%)	13.654/1.073/1.653	15.145/1.837/2.585	14.303/2.159/3.742
Limb phasing right hindlimb (%)	49.460/0.835/1.316	49.955/1.364/1.427	50.194/1.016/1.402
Maximal left metatarsal protraction (%)	5.222/2.097/2.000	4.378/2.730/4.000	2.956/1.574/2.000
Maximal right metatarsal protraction (%)	5.178/2.526/2.000	3.578/3.227/6.000	4.133/2.949/6.000
Maximal left metacarpal protraction (%)	18.144/3.662/8.000	18.600/2.890/2.000	17.256/3.336/6.000
Maximal right metacarpal protraction (%)	17.044/3.749/6.000	16.111/3.629/5.500	14.222/3.769/4.000
Maximal left metatarsal retraction (%)	18.411/1.564/2.000	18.900/2.908/2.000	16.267/3.974/6.000
Maximal right metatarsal retraction (%)	18.711/1.646/2.000	18.989/3.038/2.000	17.011/3.731/6.00
**Spatial variables**			
Sagittal ROM left hock (°)	42.093/3.278/4.761	45.462/4.304/5.500	44.121/3.567/7.841
Sagittal ROM right hock (°)	37.160/3.169/4.523	41.137/3.355/6.248	40.403/3.580/2.890
Sagittal ROM left fore cannon (°)	85.107/1.582/1.735	85.719/3.555/6.892	85.008/5.030/5.753
Sagittal ROM right fore cannon (°)	86.177/4.055/3.871	87.256/3.547/5.653	85.497/4.922/5.720
Coronal ROM mediolateral left fore cannon (°)	12.920/5.954/8.537	20.653/8.813/11.215	20.160/11.088/11.880
Coronal ROM mediolateral right fore cannon (°)	20.567/8.100/9.006	18.583/8.144/14.972	21.723/7.815/10.778
Coronal ROM mediolateral left hind cannon (°)	13.816/3.119/2.653	17.173/6.476/13.369	18.922/6.071/3.665
Coronal ROM mediolateral right hind cannon (°)	14.317/3.682/4.449	15.435/6.210/6.415	15.464/4.205/5.54
Symmetry hock	12.471/10.759/18.071	9.870/8.474/12.207	8.857/11.131/21.652

**Table 2:** Interclass correlation coefficients for each variable per surface.

	**Surface**		
**Variable**	**Treadmill**	**Soft**	**Hard**
**Temporal variables**			
Stride duration	0.998	0.993	0.999
Limb phasing left forelimb	0.989	0.991	0.990
Limb phasing right forelimb	0.993	0.992	0.996
Limb phasing right hindlimb	0.996	0.999	0.995
Maximal left metatarsal protraction	0.935	0.997	0.982
Maximal right metatarsal protraction	0.856	0.987	0.991
Maximal left metacarpal protraction	0.988	0.993	0.993
Maximal right metacarpal protraction	0.997	0.996	0.989
Maximal left metatarsal retraction	0.969	0.992	0.993
Maximal right metatarsal retraction	0.982	0.994	0.988
Mean ICC temporal variables	0.970	0.993	0.992
**Spatial variables**			
Sagittal ROM left hock	0.992	0.998	0.994
Sagittal ROM right hock	0.992	0.989	0.995
Sagittal ROM left fore cannon	0.946	0.996	0.984
Sagittal ROM right fore cannon	0.992	0.991	0.981
Coronal ROM mediolateral left fore cannon	0.999	0.999	0.998
Coronal ROM mediolateral right fore cannon	0.999	0.999	0.998
Coronal ROM mediolateral left hind cannon	0.993	0.998	0.987
Coronal ROM mediolateral right hind cannon	0.992	0.993	0.992
Symmetry hock	0.990	0.985	0.997
Mean ICC spatial variables	0.988	0.994	0.992
Mean ICC total	0.979	0.994	0.992

*Interclass correlation coefficients describe the reliability between the six different evaluators regardless of their classification (experienced, inexperienced)*.

**Table 3:** Intraclass correlation coefficients for each variable per surface.

	**Surface**		
**Variable**	**Treadmill**	**Soft**	**Hard**
**Temporal variables**			
Stride duration	0.999	0.996	0.999
Limb phasing left forelimb	0.994	0.996	0.995
Limb phasing right forelimb	0.995	0.998	0.997
Limb phasing right hindlimb	0.997	0.999	0.997
Maximal left metatarsal protraction	0.965	0.997	0.984
Maximal right metatarsal protraction	0.894	0.992	0.993
Maximal left metacarpal protraction	0.990	0.992	0.994
Maximal right metacarpal protraction	0.997	0.995	0.989
Maximal left metatarsal retraction	0.978	0.992	0.994
Maximal right metatarsal retraction	0.986	0.994	0.991
Mean ICC temporal variables	0.979	0.995	0.993
**Spatial variables**			
Sagittal ROM left hock	0.994	0.998	0.996
Sagittal ROM right hock	0.994	0.991	0.996
Sagittal ROM left fore cannon	0.953	0.998	0.987
Sagittal ROM right fore cannon	0.994	0.990	0.984
Coronal ROM mediolateral left fore cannon	0.999	0.999	0.999
Coronal ROM mediolateral right fore cannon	0.999	0.999	0.999
Coronal ROM mediolateral left hind cannon	0.995	0.998	0.989
Coronal ROM mediolateral right hind cannon	0.994	0.994	0.994
Symmetry hock	0.994	0.986	0.998
Mean ICC spatial variables	0.991	0.995	0.993
Mean ICC total	0.985	0.995	0.993

*Intraclasss correlation coefficients describe the reliability within each evaluator regardless of their classification (experienced, inexperienced)*.

Intra- and interclass correlation coefficients (ICC) were then calculated as following (22):

$$\begin{array}{l} \text{ ICC interclass}=\sigma_{\text{Horse}}^{2}/(\sigma_{\text{Horse}}^{2}+\sigma_{\text{Evaluator}}^{2}+\sigma_{\text{Repetition}}^{2})\\ \text{ICC intraclass}=(\sigma_{\text{Horse}}^{2}+\sigma_{\text{Evaluator}}^{2})/(\sigma_{\text{Horse}}^{2}+\sigma_{\text{Evaluator}}^{2}\\ \;+\sigma_{\text{Repetition}}^{2}) \end{array}$$

Whereby sigma-square is (18):

$$\begin{array}{l} \;\sigma_{\text{Horse}}^{2}=\left(\text{mean squar}e_{\text{Horse}}-{\text{mean square}}_{\text{Error}}\right)\;/\;10\\ \sigma_{\text{Evaluator}}^{2}\;=\sigma_{\text{Evaluator}}^{2}\\ \;=\left({\text{mean square}}_{\text{Evaluator}}-{\text{mean square}}_{\text{Error}}\right)/6\\ \sigma_{\text{Repetition}}^{2}={\text{mean square}}_{\text{Error}} \end{array}$$

Variance partitioning coefficients (VPC) were calculated for the factors horse, evaluator and repetition as follows, to inform on the amount of variance caused per factor (23):

$$\begin{array}{l} \text{ VPC Horse}=\sigma_{\text{Horse}}^{2}/\left(\sigma_{\text{Horse}}^{2}+\sigma_{\text{Evaluator}}^{2}+\sigma_{\text{Repetition}}^{2}\right)\\ \text{ VPC Evaluator}=\sigma_{\text{Evaluator}}^{2}/\left(\sigma_{\text{Horse}}^{2}+\sigma_{\text{Evaluator}}^{2}+\sigma_{\text{Repetition}}^{2}\right)\\ \text{VPC Repetition}=\sigma_{\text{Repetition}}^{2}/\left(\sigma_{\text{Horse}}^{2}+\sigma_{\text{Evaluator}}^{2}+\sigma_{\text{Repetition}}^{2}\right) \end{array}$$

Finally, the effect of the experience of the evaluators was assessed using a repeated measures ANOVA with horse as a subject variable, evaluator and repetition as within factors and evaluator type as a between factor. The general model repeated measures ANOVA was of the form:

$$Y_{ijkl}=\;\mu +\beta_{j}(\gamma_{k})+\;\alpha_{i}+\;\varepsilon_{ijl}$$

where *i* = 1,2,.,I, *j* = 1,2,.,J and *k* =1,2,.,K and l=1,2,…,L. Each surface was analyzed separately.

This model expresses the value of the response variable, Y, as the sum of the overall mean μ, the contribution of the *i-*th level of an explanatory variable α_*i*_ (= Evaluator ID, I = 6), the contribution of the *j-*th level of the subject variable β_*j*_ (= Horse ID, J = 10), the contribution of the *k-*th level of an explanatory variable γ_*k*_ (= EvaluatorType, K = 2) and the contribution of the *l-*th (L = 3) individual measurement ε_*ijl*_ (often called error term, *n* = 180). In the assessment, the *p*-value for the explanatory variable EvaluatorType was that of interest. Y were each of the final outcome variables presented in Tables 1–3.

## Results

In Table 1 mean, standard deviation and interquartile range for every analyzed variable were summarized and divided into the three different surfaces. The inter- and intraclass correlation coefficients were calculated and displayed in Tables 2, 3.

The correlation coefficients indicated a high reliability, and only 7% were lower than 0.98. For interclass correlation coefficients which describe the reliability between different evaluators the lowest value was 0.856 and for the intraclass correlation coefficients which define reliability within each evaluator it was 0.894. Comparing the correlation coefficients overall there was only a minimal difference in the range of values between inter and intra reliability. Mean correlation coefficients were marginally higher (differences in the third decimal) for the intra reliability on the treadmill than for on soft and hard ground. Soft surface coefficients showed the highest values followed by hard surface and then treadmill, mean ICCs ranged from 0.979 for treadmill to 0.995 for soft surface. Considering temporal and spatial variables the differences were minimal and appeared as well in the third decimal.

The ANOVA models with evaluator type showed no evidence of an effect of the experience of the evaluator in all gait variables.

Variance partitioning coefficients were highest for the factor horse with a minimum of 0.856. VPCs for the factor repetition never exceeded 0.106 and VPCs for the factor evaluator were even lower with a maximum of 0.039. Consequently this means, horse is the factor that account for the most variance in the gait analysis measurements.

## Discussion

The results achieved in this study point out overall high ICCs under the chosen conditions, this is in normal non-lame horses, which is equal to a high level of reliability of the software system. The software sensor system used to extract the data obtained by extremity mounted IMU's, which was tested here, proves to be a reliable gait analysis tool.

We chose to investigate reliability as the repeatability of this system has already been investigated (12). Reliability is the overall consistency of a measure while repeatability is the closeness of the agreement between the results of successive measurements of the same measurand carried out under the same conditions of measurement. A measure is said to have a high reliability if it produces similar results under consistent conditions (24). We were interested in documenting the correlation in between measurements made on the same subject in order to use the obtained data for remote or assisted analysis by a different person to the one doing the actual data collection.

Inter and intra correlation coefficients are the statistical tools used to define such levels of reliability and their use has been well-described previously (24, 25). The value range is defined from zero to one taking into account that an ICC of one corresponds to no measurement error and an ICC value of zero is associated with a measurement error which is responsible for the variability in measurements (24).

The comparison of the ICCs of temporal and spatial variables implies only a minimal difference in the degree of reliability. In addition none of the defined subcategories seems to show clinically relevant lower levels of reliability than the average. We believe the results of this study also allow an extrapolation for the variables which were not included in this study as they are measured or calculated in similar manner to the ones investigated and predict high levels for the ICCs.

Furthermore, whether the type of surface as an external factor has an impact on the results was evaluated. Different surfaces can have an influence on kinematic variables during a locomotion assessment (26, 27). In spite of that a reliable electronic lameness detection system should provide constant results independent of the ground situation. Measurements on the treadmill are easier to standardize after the horses have received a habituation. Important measurement conditions as speed and incline can be pre-set exactly and disturbing environmental factors can be reduced and have less influence on the horses (28). The obtained results emphasize this with equal or lower values for the SD and IQR in over 70% for treadmill comparing to soft and hard surface. Nevertheless, the mean ICCs for treadmill were lower than for soft and hard surface but with a difference smaller than 0.015 which is considered negligible. In conclusion the surface selection had no significant influence on the results, the reliability was for all the three ground situations very high.

The main benefit of this device is that it should work independently from the evaluator. If the experience of the evaluator has an influence on the results it would mean that there is no improvement compared to the visual lameness examination. To verify whether the IMU is evaluator dependent or not, variance partitioning coefficients (VPC) and probability values for the evaluator groups (experienced vs. inexperienced) were calculated. Variance partitioning coefficients are a statistical tool which is used to determine which factors are mainly responsible for any occurring variance during a measurement. The range is defined from zero to one taking into account that a value of zero means that a factor has no influence on the variance and a value of one implies that a factors is 100% responsible for the variance (29). The VPC values which were calculated focused on the factors horse (10 different horses per surface), evaluator (six people) and repetition (analysis 1, 2, and 3 made by every evaluator for all the 10 horses). The results confirm that the factor “horse” with the highest VPC values by far is mostly responsible for the variance followed by “repetition” and then “evaluator.” This might be explicable with the biological diversity and the individual locomotion pattern of every single horse. On the contrary the factors “repetition” and “evaluator” have less influence on the variation due to an apparently well-adapted analysis system with a high reliability. Whether a difference considering the reliability between the two evaluator groups, experienced and inexperienced, exists was evaluated. A measurable variation would have the consequence that for an accurate application, a prior training of the evaluator would be necessary and a major advantage of electronical gait analysis systems, evaluator independency, would be lost. During this study the probability values never reached a significant level meaning that the experience of the evaluator has no influence on the outcome. The ICCs for the intra reliability were similar to the ICCs for the inter reliability which means that the tested system works independent from the knowledge and experience of the evaluator.

Our study has some limitations. The results should be regarded critically since they can be subject to variations due to the approach of this study. Data collection was only performed with 10 non-lame horses of the same breed, but one of the main applications in the clinical field is likely to be the detection of lameness in all kind of horses. Furthermore, the data collection was done by one single person what isn't realistic in a clinical application but enabled an exact and repeatable procedure which was important in this study to minimize variations considering the results due to data collection and therefore to assess correctly any occurring variance during the analysis done by the evaluators. A further study will investigate the effect of data collection operator. On the other hand the selection of the evaluators simulated a realistic and representative situation in the clinical flied where experienced and inexperienced users will work with the tested system. Once the data has been extracted it could be sent out for interpretation by an expert. It can be criticized that not all of the gait variables were checked for reliability but with the subcategories a good system for extrapolation was found, besides it is questionable at this moment which variables are more significant for a reliable gait analysis in the field, which may vary with different applications. For example it is unknown which variables are more important for the diagnosis of lameness or the evaluation of gait quality for different disciplines.

Earlier studies have already concentrated on partly validating this inertial measurement unit system. The comparison of the IMU system with a high speed locomotion analysis system considering the metatarsal and metacarpal region in horses delivered a good agreement (18, 30). A study regarding the repeatability of gait variables obtained a high repeatability for most of the variables (12). Together with the high ICCs achieved in this study, the results received until now represent an important step for the implementation of this IMU system in the clinical application but further investigation and validation is needed.

Ideally the IMU system should be tested in a clinical environment, whereby the focus should lie especially on the use of gait analysis in lame horses of different kind of breeds and sizes. Furthermore, it would be helpful to establish a databank with data of the kinematic variables of sound and lame horses. This would establish the basis for the definition of clinical relevant ranges and assist the IMU based lameness detection. Also the importance and clinical relevance of the gait variables for lameness examination should be reviewed.

With a good initial software training the tested system is easy to use. However, it is advised to follow a strict protocol for the practical implementation of measurements to avoid bias due to the application. Important steps proved to be especially the correct placement and time stamping of the 6 sensors, repeated measurements and a correct selection of the data window for the final analysis after the data has been transferred to a computer.

In summary it can be stated that the software program used to extract data obtained by this extremity-mounted inertial measurement unit system tested in this study has a high inter- and intra-evaluator reliability under the chosen conditions and acts independent of the ground situation and the experience of the evaluator. Nevertheless, a good initial training to use the software program and to perform the measurements is essential and necessary for a correct application and a reliable gait analysis.

## Data Availability Statement

The raw data supporting the conclusions of this article will be made available by the authors, without undue reservation.

## Ethics Statement

The animal study was reviewed and approved by Animal Health and Welfare Commission of the Canton of Vaud and followed institutional guidelines for humane animal treatment (approval number VD3087; date of approval 11 February 2016).

## Author Contributions

AC conceived the study. AC, JS, MCS, MS, and UM produced the data as evaluators. JS performed the statistical analysis of the data with the help from BV. JS wrote the manuscript supported by AC and BV. AC and BV answered with equal contribution during the review process. All authors contributed to the article and approved the submitted version.

## Conflict of Interest

The authors declare that the research was conducted in the absence of any commercial or financial relationships that could be construed as a potential conflict of interest.

## Abbreviations

ICCs: Intra and inter correlation coefficients
IMU: Inertial measurement unit
ROM: Range of motion
VPC: Variance partitioning coefficient.
